# Pharmacophore screening, molecular docking, and MD simulations for identification of VEGFR-2 and c-Met potential dual inhibitors

**DOI:** 10.3389/fphar.2025.1534707

**Published:** 2025-03-07

**Authors:** Junmin Dong, Xiaohua Hao

**Affiliations:** Phase Ⅰ Clinical Trial Center, Beijing Shijitan Hospital, Capital Medical University, Beijing, China

**Keywords:** VEGFR-2, c-Met, pharmacophore, molecular docking, MD simulation

## Abstract

**Introduction:**

The vascular endothelial growth factor receptor 2 (VEGFR-2) and the mesenchymal-epithelial transition factor (c-Met) are critical in the pathogenesis and progression of various cancers by synergistically contributing to angiogenesis and tumor progression. The development of dual-target inhibitors for VEGFR-2 and c-Met holds promise for more effective cancer therapies that could overcome tumor cell resistance, a limitation often observed with inhibitors targeting a single receptor.

**Methods:**

In this study, a computational virtual screening approach involving drug likeness evaluation, pharmacophore modeling and molecular docking was employed to identify VEGFR-2/c-Met dual-target inhibitors from ChemDiv database. Subsequent molecular dynamics (MD) simulations and MM/PBSA calculations were conducted to assess the stability of the protein-ligand interactions.

**Results:**

From the virtual screening process, 18 hit compounds were identified to exhibit potential inhibitory activity against VEGFR-2 and c-Met. Among them, compound17924 and compound4312 possessed the best inhibitory potential according to our screening criteria.

**Discussion:**

The analysis of the MD simulation results indicated that compound17924 and compound4312 showed superior binding free energies to both VEGFR-2 and c-Met when compared to the positive ligands. These findings suggested that both compounds were promising candidates for further drug development and could potentially serve as improved alternatives of cancer therapeutics.

## 1 Introduction

Cancer poses a significant challenge to society, public health, and global economies in the current century. It is responsible for approximately 16.8% of all global mortalities and constitutes 22.8% of deaths associated with noncommunicable diseases (NCDs) on an international scope. According to the Global Cancer Statistics report from 2022, there were approximately 20 million new cancer diagnoses and nearly 10 million deaths due to cancer (Bray et al., 2024; Bray et al., 2021). Tumor metastasis, growth, and survival are contingent upon processes such as cellular differentiation, proliferation, angiogenesis, and apoptosis. These critical biological mechanisms are governed by an array of signaling pathways and protein kinases (Khan et al., 2023; Fan et al., 2022).

Vascular Endothelial Growth Factor (VEGF) signaling pathway stands as a crucial activator in the regulation of angiogenesis (Liu et al., 2022; Mohamed et al., 2022). VEGF receptor 2 (VEGFR-2) is the main receptor of VEGF on endothelial cells in the human body (Dorababu, 2024). When it binds to VEGF through its extracellular IgG-like domain, the tyrosine residues on the activation loop of the kinase domain undergo autophosphorylation. This initiates a series of downstream signaling pathways that mediate physiological processes such as vascular development and mitogenesis (Wang et al., 2020; Mahdy et al., 2024). Under pathological conditions, overexpression of VEGFR-2 activates the Raf-1/MAPK/ERK signaling pathway, enhances vascular permeability and is closely associated with tumor invasion and metastasis (Abdel-Mohsen et al., 2019). Therefore, inhibiting VEGFR-2 is an effective approach to hinder angiogenesis and can also combat cancer growth, proliferation, and metastasis.

The mesenchymal-epithelial transition factor (c-Met) is a transmembrane protein receptor encoded by the oncogene Met and belongs to receptor tyrosine kinase (RTK) family (Michaelides et al., 2023; Uchikawa et al., 2021). Upon specific binding of hepatocyte growth factor (HGF) to c-Met in its extracellular domain, the conformation of the c-Met receptor protein is altered, leading to the activation of the protein tyrosine kinase (PTK). This activation facilitates the dimerization and transphosphorylation of tyrosine residues Tyr1234 and Tyr1235, initiating downstream signaling phosphorylation reactions (Zhang et al., 2022; Comoglio et al., 2008). Through a series of cascade amplification effects, it can regulate transcription and translation within the nucleus, thereby completing the process of cell proliferation (Mer et al., 2024; Sun et al., 2024). Overexpression of HGF/SF, abnormal expression of c-Met, and mutation activation, and so on, can lead to abnormal activation of the c-Met signaling pathway, thereby promoting abnormal proliferation, growth, invasion, and diffusion of tumor cells (Zhang et al., 2020; Maria et al., 2019). Therefore, inhibiting the abnormal activation of the c-Met signaling pathway has emerged as a promising approach in cancer therapy.

Drug combinations or single chemical entity with different mechanisms can block diverse cancer pathways by targeting multiple factors in complex diseases, potentially producing positive outcomes in cancer treatment (Gilad et al., 2021; Coen van Hasselt and Iyengar, 2019). VEGFR-2/VEGF and c-Met/HGF are both overexpressed in many human cancers and have a synergistic effect in the progression of numerous diseases (Gu et al., 2017; Sun et al., 2024). Consequently, VEGFR-2/c-Met dual inhibitors may offer broader benefits compared to selective inhibitors targeting either VEGFR-2 or c-Met in various malignancies. In recent years, the research of VEGFR-2 and c-Met dual inhibitors has also become more and more extensive. The skeletons of them mainly include pyridine, quinoline, pyrrolopyridine, benzimidazole, thienopyrimidine, and pyrrolotriazine (Carvalho et al., 2021). Cabozantinib was the first c-Met/VEGFR-2 dual-target inhibitor developed by Exelixis (Deeks, 2019). It was approved by the US FDA in 2012 for the treatment of metastatic medullary thyroid cancer. And some compounds such as foretinib (Zillhardt et al., 2011), golvatinib (Nakagawa et al., 2010), BMS-794833 (Nishida-Aoki et al., 2022), and dovitinib (Semrad et al., 2017) have entered into clinical trials.

To search potential c-Met/VEGFR-2 dual target inhibitors with novel structures, a comprehensive virtual screening approach that integrated pharmacophore modeling, molecular docking, and MD simulation was employed. Initially, 1.28 million compounds were filtered based on Lipinski and Veber rules, followed by ADMET (absorption, distribution, metabolism, excretion, and toxicity) predictions. Subsequently, a set of pharmacophores for VEGFR-2 and c-Met proteins were developed and assessed, and the best two pharmacophores were selected for molecular database screening. Then the compounds were docked with VEGFR-2 and c-Met targets to identify those with superior binding affinities. Finally, 2 hit compounds were chosen for 100 ns MD simulations to assess their binding stability.

## 2 Materials and methods

### 2.1 Preparation of proteins

**FIGURE 1:**
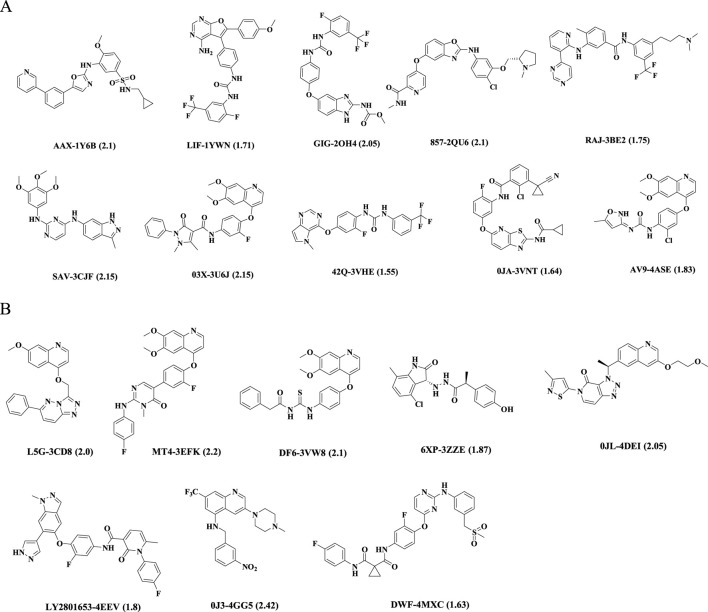
Chemical structures of ligands in PDB files. Ligand name-PDB ID (resolution, Å). **(A)** VEGFR-2 inhibitors, **(B)** c-Met inhibitors.

18 co-crystal structures of VEGFR-2 and 47 co-crystal structures of c-Met were obtained from RCSB Protein Data Bank website (http://www.rcsb.org/). Considering a resolution of less than 2 Å, biological activity at the nm level, diversity of structures and others, 10 VEGFR-2 complexes and 8 c-Met complexes were ultimately selected for building pharmacophore models. Supplementary Tables S1, S2 provided detailed information about them. The 2D structures of corresponding ligands were shown in Figure 1. All the protein structures were prepared in the Discovery Studio 2019 software (hereinafter referred to as DS, 2019) (Yang et al,. 2020), which included removing water molecules in the PDB files, completing the missing amino acid residues, correcting the connectivity and order of bonds, and minimizing the energy of the complexes using the CHARMM force field.

### 2.2 Preparation of ligands

In this study, VEGFR-2 validation set with 400 compounds was constructed. Among them, 25 VEGFR-2 inhibitors were collected from various publications and 375 inactive compounds were downloaded from DUD-E website (https://dude.docking.org/) (Mysinger et al., 2012). Here, active compounds referred to those with the experimental activity less than 0.01 μM, inactive compounds refer to those druglike compounds, whereas with unknown pharmacological action or against different proteins other than VEGFR-2. c-Met validation set with 25 c-Met inhibitors and 400 inactive compounds was collected as the same way. All decoy sets were prepared by Prepare Ligands protocol in DS (2019) software.

### 2.3 Pharmacophore generation and evaluation

Based on the above crystal structure, the pharmacophores were constructed using the Receptor Ligand Pharmacophore Generation module in DS (2019) software. In this procedure, the Maximum Pharmacophores were set to 10. Six standard pharmacophore features: hydrogen bond acceptor, hydrogen bond donor, positive ionizable center, negative ionizable center, hydrophobic center, and ring aromatic center were considered. The minimum and maximum features were set to 4 and 6, respectively. Other parameters were kept at default settings. Then, decoy sets were used to validate the ability of generated pharmacophores enriching active compounds from inactive compounds. Enrichment factor (EF) value and AUC value, the area under receiver operating characteristic (ROC) curve were calculated to assess the quality of the pharmacophore model (Dong and Wang, 2023). EF value was defined as follows:

$$\text{EF}=\left(\text{Ha }\times D\right)/\left(\text{Ht}\times \;A\right)$$

where Ha was total number of active compounds that were identified as hits through pharmacophore-based screening; D was total number of compounds in decoy set; Ht was total number of active compounds from the decoy set; A was total number of compounds returned by pharmacophore-based screening.

In general, a model is considered reliable if it has an AUC greater than 0.7 and an EF value exceeding 2 (Zhang et al., 2023). Considering these criteria, the pharmacophores for VEGFR-2 and c-Met with the top enrichment factor (EF) values were selected for the virtual screening process.

### 2.4 Drug likeness filtration

More than 1.28 million compounds were collected from commercial ChemDiv database (Topscience, Shanghai, China). All counterions, solvent moieties, and salts were removed from their structures, and the hydrogen atoms were added. The database was firstly screened by Lipinski and Veber rules in Prepare or Filter Ligands protocol in DS (2019) software. ADMET (absorption, distribution, metabolism, excretion, and toxicity) descriptors were then applied to check whether the compounds had the properties of aqueous solubility, blood brain barrier penetration (BBB), cytochrome P4502D6 inhibition, hepatotoxicity, human intestinal and plasma protein binding in the Calculate Molecular Properties protocol. The cut-off value of the solubility, absorption, and the BBB were 3, 0, and 3, respectively (Huang et al., 2021).

### 2.5 Pharmacophore screening

Firstly, validated VEGFR-2 pharmacophore served as 3D queries to identify potent compounds among the above remaining molecules. The Fast search method in Ligand Pharmacophore Mapping protocol was employed to screen these compounds and the other parameters remained default. Compounds with fit value exceeding 2.4 were retained and then compiled into a new smaller molecular database. This refined database permitted 255 conformations for each compound and subsequently underwent a secondary screening utilizing the c-Met pharmacophore. Similarly, compounds with fit value above 2.4 were selected for further molecular docking calculations.

### 2.6 Molecular docking

Molecular docking served as a computational strategy to identify binding mode and affinity of molecules with receptors. In this study, the crystal structure of VEGFR-2 (PDB ID: 2OH4) in a complex with compound GIG and c-Met (PDB ID: 3EFK) in a complex with MT4 were utilized as the basis for molecular docking (Hasegawa et al., 2007; D’Angelo et al., 2008). This was achieved through the application of both Libdock and CDOCKER modules within DS (2019) software. The original ligands of the protein structures were used to define the binding sites. RMSD values between the redocked and the original conformations were calculated. A value below 2 Å was considered reliable for docking results (Wang et al., 2020). Subsequently, compounds selected by pharmacophores were docked into the binding pocket using the LibDock protocol with standard parameters, except for calculating ligand conformations within an energy range of 10 kcal⋅mol^−1^. Compounds with high LibDock score were subjected to refined docking using CDOCKER protocol.

### 2.7 MD simulation and binding free energy calculation

MD simulation typically studied the dynamic behavior and temporal evolution characteristics of protein and ligand systems (Bai et al., 2023; Zeb et al., 2019). In this research, GROMACS version 2018.8 was employed to run MD simulation upon CHARMM36 force field (Matada et al., 2022). Firstly, protein topologies were derived from the pdb2gmx tool and ligand topologies were obtained through the CgenFF server (Tondar et al., 2024). Upon merging, they formed the essential topology files for each protein-ligand complex. Secondly, these complexes were situated at the center of a cubic simulation box, maintaining a distance of 4.0 nm from the edges to the protein exterior. Then, the system was hydrated with TIP3P water model, and two Cl^−^ ions were introduced to neutralize additional charge. Subsequently, the process of energy minimization was undertaken through 50,000 steps of the steepest descent method. 100 ps NVT (constant number of particles, volume, and temperature) equilibration and 1,000 ps NPT (constant number of particles, pressure, and temperature) equilibration were performed. After equilibrium, the system underwent conventional MD simulation with a time step of 2 fs and a duration of 100 ns. The trajectories were recorded every 10 ps. Root mean square deviation (RMSD), root mean square fluctuation (RMSF), and radius of gyration (Rg) values were calculated to assess the stability and flexibility of the complexes throughout the simulation duration. Finally, the binding energies of the complexes were computed using gmx_MM/PBSA tool. The method relied on molecular mechanics/Poisson-Boltzmann surface area (MM/PBSA) calculation to quantify protein-ligand binding free energy, offering insights into ligand affinity (Fan et al., 2024).

## 3 Results and discussion

### 3.1 Pharmacophore generation and evaluation

Each protein complex generated 6–10 pharmacophores and corresponding calculated parameters were detailed in Supplementary Table S3. The pharmacophore with the highest enrichment factor (EF) value from each group was selected. As shown in Table 1, the range of EF values for the top ten VEGFR-2 pharmacophores was between 1.67 and 15.3. Among them, the EF values for 2OH4 01, 2QU6 01, 3BE2 06, 3U6J 06, 3VNT 06, and 4ASE 07 exceed 10.0, indicating their high specificity towards VEGFR-2 inhibitors. Pharmacophore 2OH4 01 had the maximum EF value and contained six features, including three hydrogen bond donors (D), and three hydrophobic centers (H). The results suggested that the two features played significant roles in the binding of VEGFR-2 inhibitors to the protein. Similarly, the EF values computed by the top eight pharmacophores targeting c-Met varied between 1.8 and 15.3 (Table 2). And model 3EFK 01 was the only pharmacophore with an EF value exceeding 10.0 (EF = 15.3). It incorporated six features including a hydrogen bond acceptor (A), four hydrophobic centers (H), and a ring aromatic center (R).

**TABLE 1:** The results for the top ten VEGFR-2 pharmacophores.

VEGFR-2 Pharmacophore	Ht	A	EF	AUC	Features^a^
1Y6B 01	25	197	1.67	0.761	AHHHH
1YWN 03	7	15	7.47	0.629	ADDDHH
2OH4 01	22	23	15.3	0.940	DDDHHH
2QU6 01	19	26	11.69	0.873	ADDHHH
3BE2 06	8	10	12.8	0.658	AHHHPR
3CJF 01	25	239	1.67	0.835	ADHHH
3U6J 06	5	8	10	0.596	HHHHHH
3VHE 10	9	15	9.6	0.673	ADDHHH
3VNT 06	20	25	12.8	0.897	DDHHHH
4ASE 07	21	28	12	0.913	DDHHHH

^a^ A: hydrogen bond acceptor, D: hydrogen bond donor, H: hydrophobic center, P: positive charge center, R: ring aromatic center.

**TABLE 2:** The results for the top eight c-Met pharmacophores.

c-Met Pharmacophore	Ht	A	EF	AUC	Features^a^
3CD8 01	25	199	2.01	0.795	AHHHH
3EFK 01	23	24	15.3	0.960	AHHHHR
3VW8 06	18	47	6.13	0.832	AAAHHH
3ZZE 01	25	222	1.80	0.725	AAHHH
4DEI 01	24	115	3.34	0.889	AAHHHH
4EEV 07	11	21	8.38	0.709	AHHHHR
4GG5 10	16	69	3.71	0.729	ADHHHR
4MXC 09	9	25	5.76	0.661	AADHHR

^a^ A: hydrogen bond acceptor, D: hydrogen bond donor, H: hydrophobic center, R: ring aromatic center.

**FIGURE 2:**
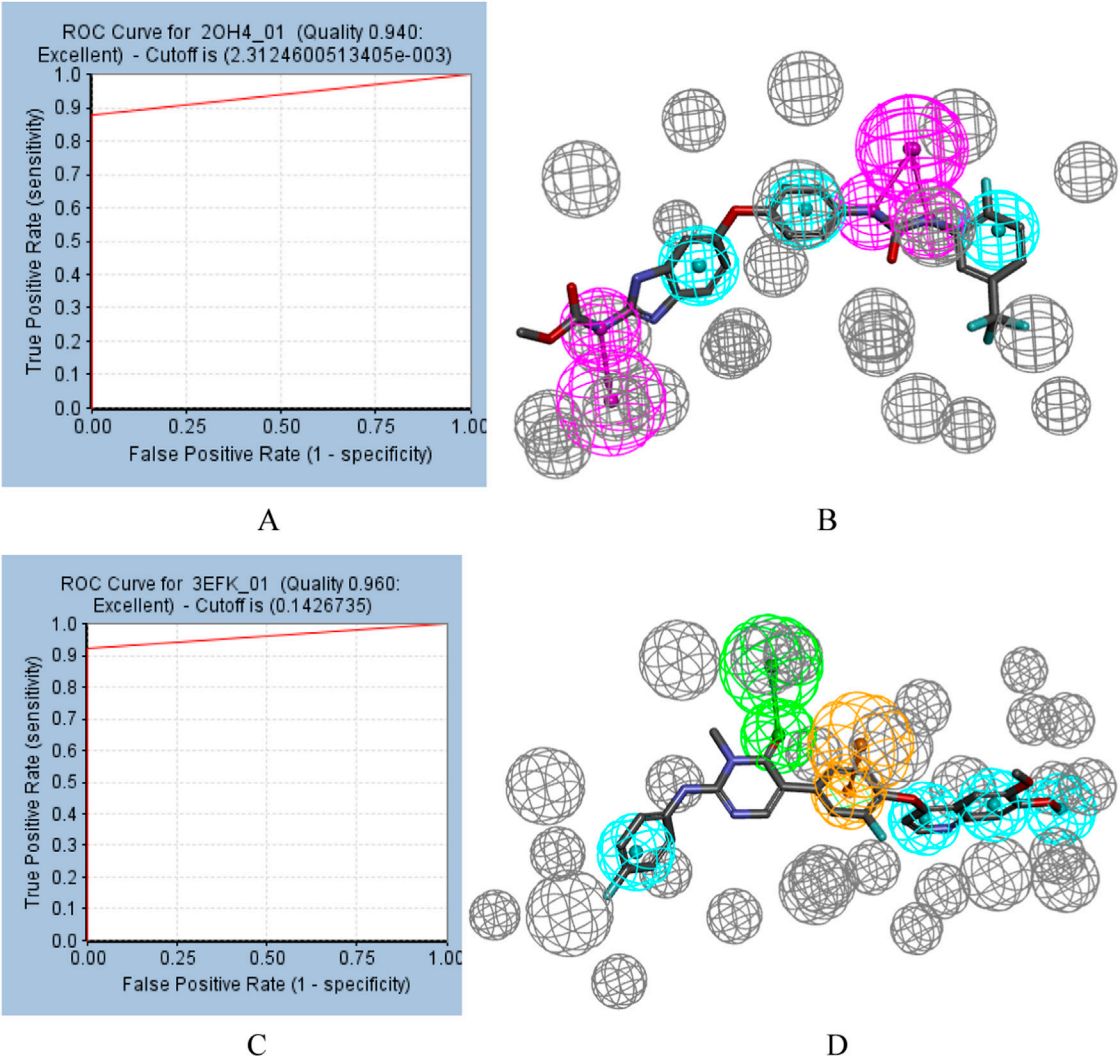
ROC curves for pharmacophore **(A)** 2OH4 01 and pharmacophore **(C)** 3EFK 01. Pharmacophore 2OH4 01 **(B)** and pharmacophore 4EFK 01 **(D)** with original ligands. Gray balls were for excluded volumes, purple for a hydrogen bond donor, blue for a hydrophobic center, orange for a ring aromatic center, and green for a hydrogen bond acceptor.

ROC curve also played an important role in the evaluation of pharmacophores. ROC curves for pharmacophore 2OH4 01 and pharmacophore 3EFK 01 were shown in Figure 2. And their AUC values were 0.940 and 0.960, respectively, indicating their ability to distinguish active and inactive compounds. Thus, the two pharmacophores (2OH4 01 and 3EFK 01) were selected for subsequent analysis.

### 3.2 Parameter setting for molecular docking

**FIGURE 3:**
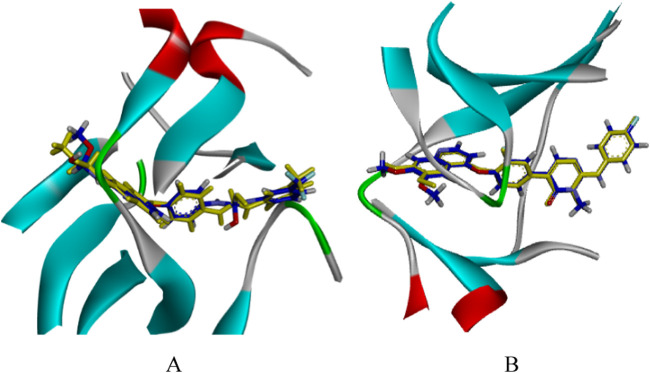
The CDOCKER docking mode of compound GIG to VEGFR-2 [PDB: 2OH4, **(A)**]. The CDOCKER docking mode of compound MT4 to c-Met [PDB: 3EFK, **(B)**]. Yellow: the original conformation; Blue: the conformation after docking.

The docking parameters were pivotal for the effectiveness of virtual screening (Bender et al., 2021). We optimized them in advance by using the original ligand GIG and MT4 as references. The docking parameters were adjusted to align the docked conformations closely to the original conformations. Ultimately, the radii of the spherical box were set to 12.5 Å for VEGFR-2 and 12 Å for c-Met, respectively. Figure 3 demonstrated the superimposition of the original X-ray crystal ligands (depicted in yellow sticks) with the conformations obtained from CDOCKER protocol (shown in blue sticks), revealing a slight deviation in the branched chain of GIG and MT4. And the RMSD values of docking conformations were 0.8199 Å and 0.2934 Å, respectively. These findings validated the reliability of docking parameters for subsequent virtual screening.

### 3.3 Virtual screening

**FIGURE 4:**
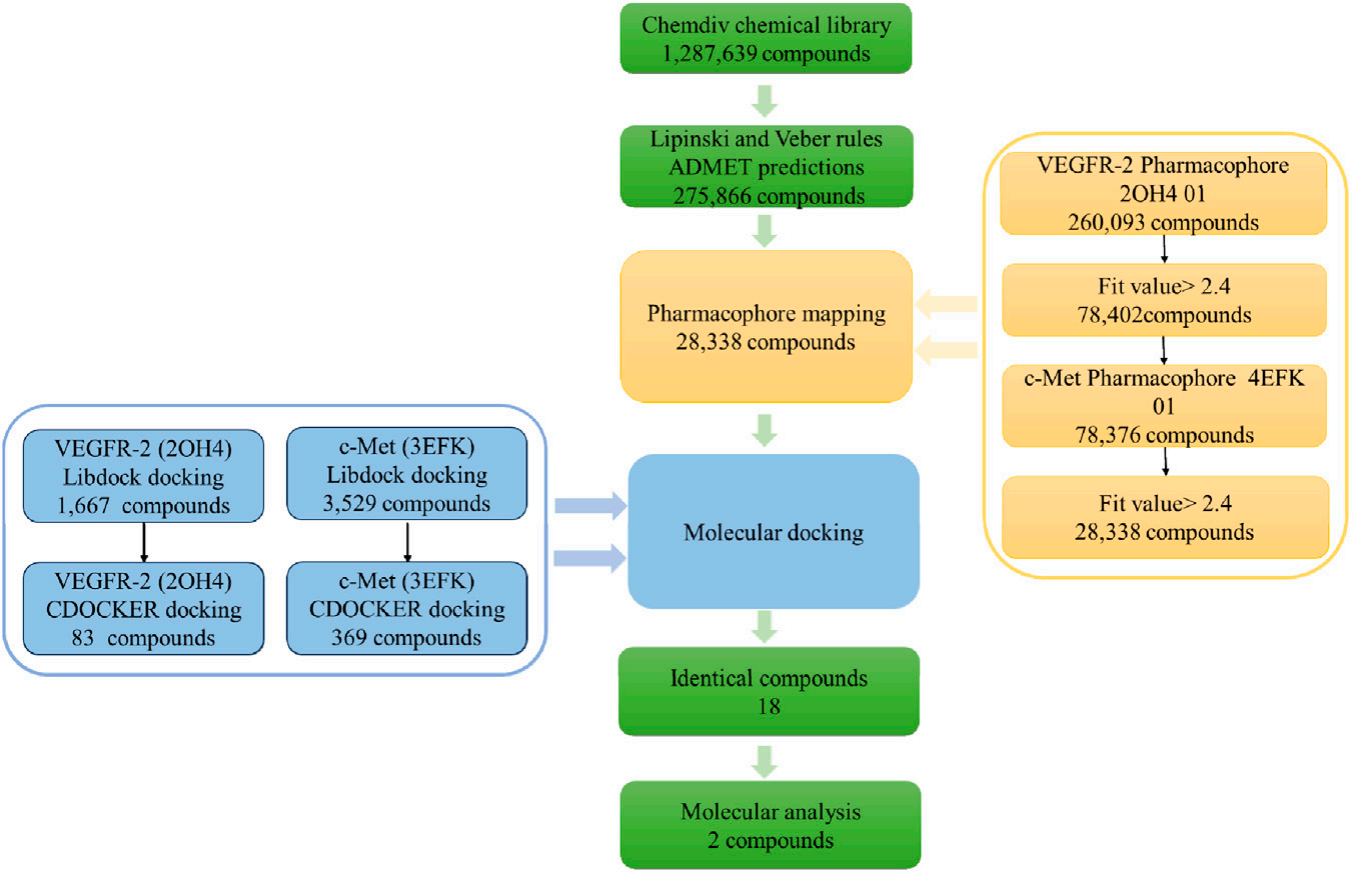
The workflow of virtual screening.

The workflow of screening procedure was shown in Figure 4. Firstly, a chemical library from the ChemDiv database, encompassing 1.28 million commercially available compounds was screened by using Lipinski and Veber rules, and ADMET predictions. Out of these, a total of 275,866 compounds fulfilled the set criteria.

Next, the remaining compounds were further screened using the optimally validated pharmacophores 2OH4 01 and 4EFK 01 as 3D queries. The initial screening stage employed the pharmacophore 2OH4 01, resulting in 260,093 compounds that aligned with all its features, of which 78,402 exhibited fit values greater than 2.4. These selected compounds were then compiled into a new, refined molecular database for a second round of screening utilizing the pharmacophore 4EFK 01. And a subset of 28,338 compounds that achieved fit values exceeding 2.4 were identified for molecular docking.

Subsequently, molecular docking was employed to assess the potential interactions between these compounds with their target proteins. Utilizing the LibDock protocol, we ranked the binding affinities of 28,338 compounds to VEGFR-2 and retained 1,667 compounds with LibDock scores above 142.5. In a similar way, 3,529 compounds targeting c-Met were identified with LibDock scores exceeding 142.6. These selected molecules were further docked with the VEGFR-2 or c-Met receptors using the CDOCKER protocol. Out of these, 83 compounds demonstrated a superior CDOCKER_INTERACTION_ ENERGY to the original ligand GIG, and 369 compounds surpassed MT4. By comparison, there were a total of 18 identical molecules. Their structures and virtual screening results were shown in Supplementary Table S4. The selected compounds demonstrated significant binding affinity for VEGFR-2 and c-Met, achieving docking scores below −60 kJ⋅mol^−1^. The results indicated that each of the chosen compounds exhibited a stronger affinity for VEGFR-2 and c-Met when compared to the original ligands. Compound17924 (3-(2-(aminomethyl)-5,7-dimethyl-[1,2,4] triazolo[1,5-a]pyrimidin-6-yl)-N-((1-(2,5-dimethylbenzyl) piperidin-4-yl)methyl) propanamide) and compound4312 (3-(3-(4-(2-methoxyethoxy)phenyl)-5-oxo-2,5-dihydro-1,2,4-triazin-6-yl)-N-(2-(pyridin-2-yl)ethyl)propanamide) had the highest docking scores for both two receptors and were selected for further analysis.

### 3.4 Analysis of binding mode

**FIGURE 5:**
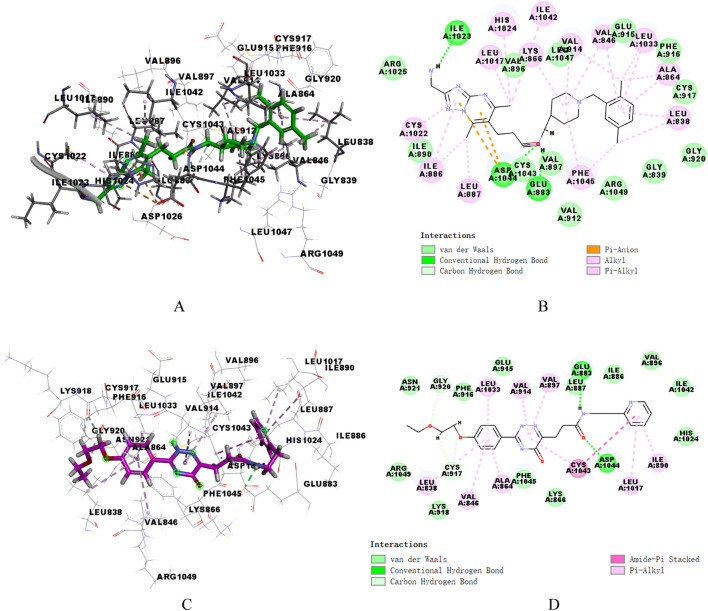
3D and 2D binding mode of compound17924 **(A, B)** and compound4312 **(C, D)** into VEGFR-2 active site.

Ligand Interaction diagram in DS (2019) software was utilized to analyze the patterns of intermolecular interactions between the two proteins and two hit compounds. Figure 5 presented the interactions of two compounds with VEGFR-2. Compound17924 engaged the linker region via its amide moiety, establishing hydrogen bonds with amino acid residues Glu883 and Asp1044, and formed numerous hydrophobic interactions with Cys1043, Val912, and Val897. Then, compound17924 was anchored into the active site of VEGFR-2 protein by pivotal hydrogen bond interactions between its terminal amino group and Ile1023. Additionally, the [1,2,4] triazolo [1,5-a] pyrimidine moiety nestled into the hydrophobic pocket, contributing pi-anion interactions with the amino acid Asp1044. The 1-benzylpiperidine segment contributed to a host of alkyl, pi-alkyl, and hydrophobic interactions engaging hinge region residues like Leu838, Ala864, Cys917, among others. The binding mode of compound4312 within VEGFR-2 was similar to that of compound17924. The hydrogen bond interactions of the amino and carbonyl groups within the amide moiety with Glu883 and Asp1044 were maintained. In addition, the pyridine ring and the 5-oxo-2,5-dihydro-1,2,4-triazin segment of compound4312 achieved stabilization within the binding pocket through aromatic stacking interactions with the residue Cys1043. The phenyl side chain was accommodated in the hydrophobic cavity shaped by the residues Glu915, Phe916, Lys918, Gly920, and Arg1049.

**FIGURE 6:**
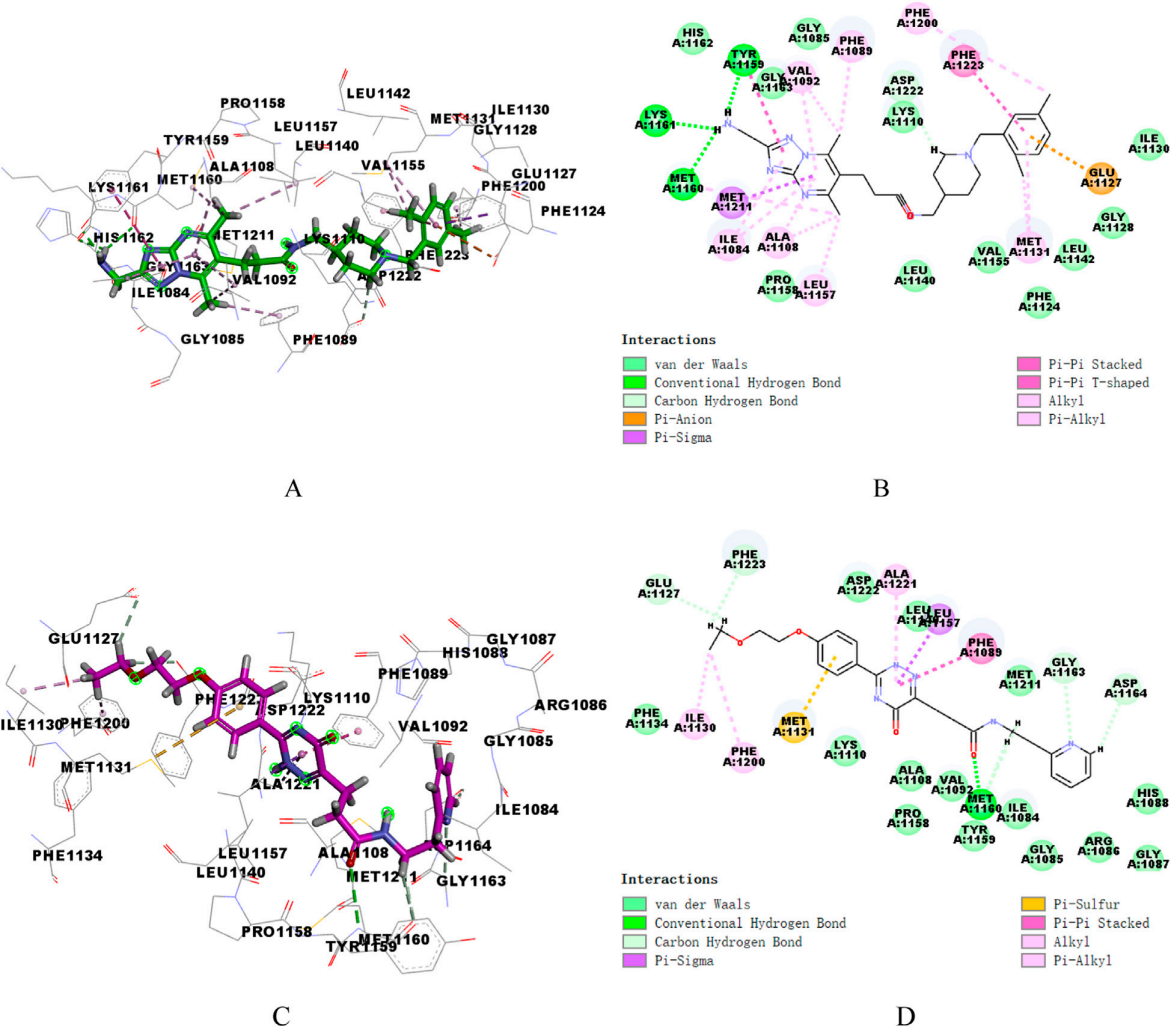
3D and 2D binding mode of compound17924 **(A, B)** and compound4312 **(C, D)** into c-Met active site.

Similarly, the docking patterns for binding of compounds 17,924 and 4,312 to c-Met were presented in Figure 6. The terminal amino group of compound17924 participated in hydrogen bonding with the residues Tyr1159, Met1160, and Lys1161 within the active site of c-Met (Figures 6A, B). The [1,2,4] triazolo [1,5-a] pyrimidine segment of compound17924 was anchored through pi-sigma interactions with Met1211, alongside numerous pi-alkyl and alkyl interactions. Pronounced hydrophobic contacts were noted with residues such as Lys1110, Phe1124, Gly1128, Ile1130, Leu1140, Leu1142, Val1155, and Ala1221. Residing within the active site of c-Met, compound4312 engaged in various interactions including hydrogen bonds, pi-pi stacking, pi-sigma, and hydrophobic interactions with adjacent amino acids, as illustrated in Figures 6C, D. The amide group formed hydrogen bonds with Met1160, while its 5-oxo-2,5-dihydro-1,2,4-triazin segment participated in pi-pi stacking and pi-sigma interactions with Phe1089 and Leu1157. Compound4312 also established hydrophobic contacts with residues like Ile1084, Gly1085, Arg1086, Gly1087, His1088, and Asp1164. Notably, the phenyl group entered into a pi-sulfur interaction with the sulfur-bearing side chain of Met1131. Overall, these crucial interactions suggested that both compound17924 and compound4312 could engage effectively with key residues within VEGFR-2 and c-Met, indicating their potential to inhibit target proteins.

### 3.5 MD simulation

**FIGURE 7:**
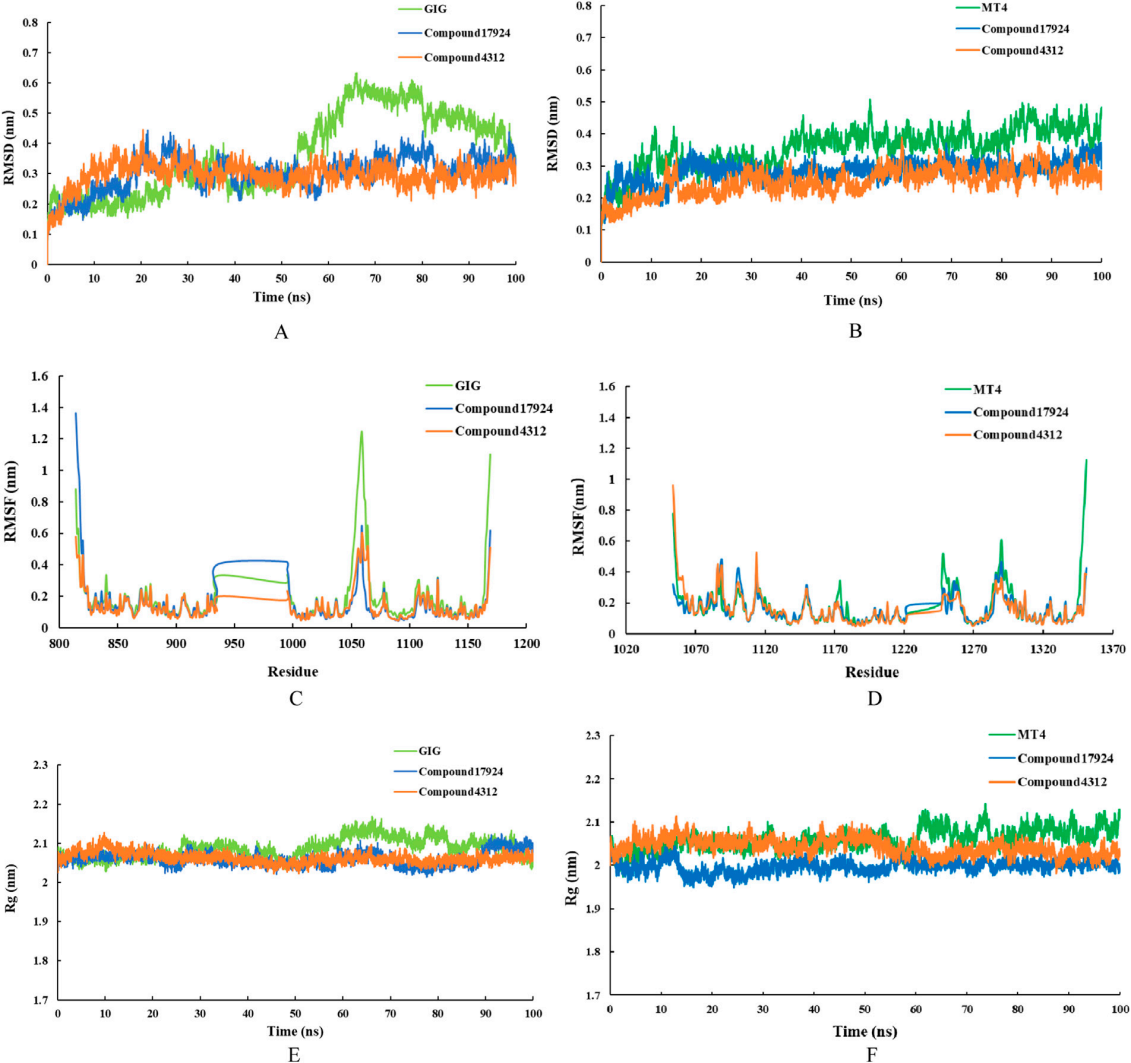
MD simulation results for the interactions of compound17924 (depicted in blue) and compound4312 (shown in orange) with VEGFR-2 (illustrated in the left panel) and c-Met (depicted in the right panel) were presented. The original ligand GIG (shown in light green) and MT4 (shown in dark green) were as references. The studies included the time-dependent RMSD of the backbone atoms for **(A)** VEGFR-2 complexes and **(B)** c-Met complexes, RMSF of the backbone atoms for **(C)** VEGFR-2 complexes and **(D)** c-Met complexes, as well as time-evolving Rg values of the backbone atoms for **(E)** VEGFR-2 complexes and **(F)** c-Met complexes.

The RMSD values of the backbone atoms within a complex were utilized to assess the equilibrium stability during the simulation period. A low RMSD value represented a stable protein-ligand system (Yu et al., 2024). To evaluate the dynamic stability of the potential inhibitors in association with VEGFR-2 and c-Met kinases, 100 ns MD simulations were conducted on the respective complexes: VEGFR-2 with GIG, VEGFR-2 with compound17924, VEGFR-2 with compound4312, c-Met with MT4, c-Met with compound17924, and c-Met with compound4312. Figures 7A, B illustrated the temporal fluctuations in RMSD values for the backbone atoms across the six complexes. According to Figure 7, the RMSD values of VEGFR-2-GIG complex showed significant fluctuations in the latter half (55ns–100 ns), with a peak RMSD reaching 0.6 nm. In contrast, compound17924 and compound4312 demonstrated superior stability upon binding to the system. The RMSD values of both complexes showed an increase during the early simulation stages, but ultimately stabilized around the 40 ns, maintaining an average fluctuation around 0.3 nm. Figure 7B showed that the RMSD trajectories of all three c-Met complexes displayed similar fluctuations within the initial 35 ns, settling into stability thereafter. The mean RMSD values for the c-Met-MT4, c-Met-compound17924, and c-Met-compound4312 complexes were 0.3568, 0.2834, and 0.2498 nm, respectively (Supplementary Table S5). These values implied that compound17924 and compound4312 might form more favorable interactions with c-Met than MT4. And compared with the c-Met-compound17924 complex, the c-Met-compound4312 complex demonstrated greater stability.

Following these, the RMSF values for the residues in the system at equilibrium were evaluated. RMSF values typically illustrated the variances among individual residues along the protein sequence, serving as a measure of the protein-ligand structural flexibility (Zhai et al., 2021). In this case, an increase in RMSF values would suggest an increase in fluctuations. Figures 7C, D depicted that the extent of fluctuations for Chain A residues across the six different systems was broadly comparable, indicating that the binding modes of the ligands with either VEGFR-2 or c-Met kinase remained consistent. As shown in Figure 7C, the RMSF values for certain amino acids, such as Glu883, Ile1023, and Asp1044 in VEGFR-2-compound17924 and VEGFR-2-compound4312 complexes were reduced in comparison to those of VEGFR-2 with GIG. This suggested that compound17924 and compound4312 established more robust interactions with these specific residues, which aligned with the outcomes of the preceding molecular docking analyses. Similarly, the essential residues in all c-Met-related complexes displayed minimal flexibility, implying that the principal interactions of all compounds within the binding pocket might be comparable.

The Rg value was a measure of the overall compactness of the system (Bagewadi et al., 2023). The curves in Figures 7E, F suggested that the protein structures of all complexes remained essentially stable. The VEGFR-2 complexes experienced minor fluctuations before their Rg values settled within the range of 2.05–2.1 nm. Similarly, the Rg values for all c-Met complexes eventually stabilized between 1.95 and 2.1 nm. Upon comparing the protein complexes bound to the original ligands (GIG and MT4) with those bound to compound17924 and compound4312, it was observed that Rg values for the protein backbone achieved equilibrium at comparatively lower levels in the latter. These results implied that the interaction of compound17924 and compound4312 with protein contributed to the enhanced stabilization of the system.

To assess the binding affinities of the compounds within the six systems, their binding free energies were determined utilizing the MM/PBSA method. As shown in Table 3, the binding free energies of GIG, compound17924, compound4312 in VEGFR-2 were −88.638, −134.072, −94.904 kJ⋅mol^−1^, respectively, and in c-Met were −23.696, −118.564, −81.097 kJ⋅mol^−1^, respectively. Compound17924 and compound4312 exhibited higher binding affinities compared to the original compounds GIG and MT4. And compound17924 might have the strongest binding ability. For three VEGFR-2 complexes, the polar solvation energy, SASA energy, and Van der Waals energy remained relatively consistent. The electrostatic energy of VEGFR-2-compound17924 complex was notably lesser in comparison to that of GIG and compound4312, which might account for its superior binding free energy. For c-Met complexes, although the compound17924 and compound4312 exhibited relatively high polar solvation energies, their van der Waals and electrostatic energies were lower, which made significant contributions to their overall binding affinities. Based on these analyses, the identified compound17924 and compound4312 could potentially be candidates for VEGFR-2/c-MET dual-target inhibitors.

**TABLE 3:** Binding free energies of the screened compounds.

Complex	Polar solvation energy (kJ⋅mol^−1^)	SASA energy (kJ⋅mol^−1^)	Electrostatic energy (kJ⋅mol^−1^)	Van der Waals energy (kJ⋅mol^−1^)	Binding free energy (kJ⋅mol^−1^)
VEGFR-2-GIG	225.980	−30.231	−57.255	−227.133	−88.638
VEGFR-2-Compound17924	269.471	−29.808	−166.051	−207.684	−134.072
VEGFR-2-Compound4312	210.689	−24.850	−71.133	−209.610	−94.904
c-MET-MT4	146.678	−24.005	25.556	−171.925	−23.696
c-MET-Compound17924	223.239	−30.939	−52.294	−258.570	−118.564
c-MET-Compound4312	233.053	−28.951	−55.909	−229.290	−81.097

## 4 Conclusion

Inhibitors simultaneously targeting c-Met and VEGFR-2 are regarded as an encouraging strategy for cancer therapy. The objective of this research was to discover potential VEGFR-2/c-Met dual-target inhibitors through virtual screening of the ChemDiv database. Initially, pharmacophore models were constructed based on receptor-ligand complexes, and these models underwent validation using decoy sets. The two optimal pharmacophores were subsequently chosen for screening the database. Additionally, assessments of drug likeness were performed to identify compounds with favorable safety profiles. Following these steps, molecular docking was conducted to obtain compounds that demonstrated strong binding affinities. Ultimately, 18 identical compounds were identified. Among them, compound17924 and compound4312 emerged with the superior docking scores against both VEGFR-2 and c-Met. To further study the binding stability of compound17924 and compound4312 with two receptors, 100 ns MD simulations were executed to examine the stability of the complex systems. Analyzing of RMSD, RMSF, and Rg values suggested the two hit compounds had stronger stability. In conclusion, our study indicated that compound17924 and compound4312 were promising candidates as safe and effective VEGFR-2/c-MET dual-target inhibitors. This work would lay the foundation for the development of anti-tumor drugs.

## Data Availability

The original contributions presented in the study are included in the article/Supplementary Material, further inquiries can be directed to the corresponding author.
