# Supplementary material for: Pharmacophore screening, molecular docking, and MD simulations for identification of VEGFR-2 and c-Met potential dual inhibitors
Source: Front Pharmacol. 2025 Mar 7;16:1534707. doi: 10.3389/fphar.2025.1534707 (PMC11926154; doi:10.3389/fphar.2025.1534707)
Supplement: Supplementary file 1 [file Table1.docx]

Supplementary Material

# Supplementary Tables

**Supplementary Table 1.** Detailed information about co-crystal structures of VEGFR-2 with inhibitors.

| PDB | Organism(s) | Released date | Method | Resolution | Literature | Active data (IC_50_) |
| --- | --- | --- | --- | --- | --- | --- |
| 1Y6B | Homo sapiens | 2005-06-07 | X-RAY | 2.1 Å | J Med Chem, 2005, 48: 1610-1619 | 38 nM |
| 1YWN | Homo sapiens | 2005-08-23 | X-RAY | 1.71 Å | Bioorg Med Chem Lett, 2005, 15: 2203-2207 | 15.39 nM |
| 2OH4 | Homo sapiens | 2007-09-18 | X-RAY | 2.05 Å | J Med Chem, 2007, 50: 4453-4470 | 3.5 nM |
| 2QU6 | Homo sapiens | 2007-09-25 | X-RAY | 2.1 Å | J Med Chem, 2007, 50: 4351-4373 | 4.6 nM |
| 3BE2 | Homo sapiens | 2008-04-08 | X-RAY | 1.75 Å | J Med Chem, 2008, 51: 1649-1667 | 2 nM |
| 3CJF | Homo sapiens | 2008-10-07 | X-RAY | 2.15 Å | J Med Chem, 2008, 51: 4632-4640 | 6.3 nM |
| 3U6J | Homo sapiens | 2012-02-22 | X-RAY | 2.15 Å | J Med Chem, 2012, 55: 1858-1867 | 6.2 nM |
| 3VHE | Homo sapiens | 2011-11-02 | X-RAY | 1.55 Å | Bioorg Med Chem, 2010, 18: 7260-7273 | 6.2 nM |
| 3VNT | Homo sapiens | 2012-04-11 | X-RAY | 1.64 Å | J Med Chem, 2012, 55: 3452-3478 | 2.2 nM |
| 4ASE | Homo sapiens | 2012-09-26 | X-RAY | 1.83 Å | Proc Natl Acad Sci U S A, 2012, 109: 18281 | 0.01 nM |

**Supplementary Table 2.** Detailed information about co-crystal structures of c-Met with inhibitors.

| PDB | Organism(s) | Released date | Method | Resolution | Literature | Active data (IC_50_) |
| --- | --- | --- | --- | --- | --- | --- |
| 3CD8 | Homo sapiens | 2008-04-29 | X-RAY | 2 Å | J Med Chem, 2008, 51: 2879-2882 | 9 nM |
| 3EFK | Homo sapiens | 2008-10-07 | X-RAY | 2.2 Å | J Med Chem, 2008, 51: 5766-5779 | 14 nM |
| 3VW8 | Homo sapiens | 2013-08-14 | X-RAY | 2.1 Å | Bioorg Med Chem, 2013, 21: 7686-7698 | 2 nM |
| 3ZZE | Homo sapiens | 2011-09-14 | X-RAY | 1.87 Å | J Med Chem, 2012, 55: 8091 | 1.3 nM (Ki) |
| 4DEI | Homo sapiens | 2012-05-30 | X-RAY | 2.05 Å | Bioorg Med Chem Lett, 2012, 22: 4089-4093 | 1 nM |
| 4EEV | Homo sapiens | 2013-04-10 | X-RAY | 2.1 Å | Invest New Drugs, 2013, 31: 833-844 | 1 nM |
| 4GG5 | Homo sapiens | 2012-10-03 | X-RAY | 2.423 Å | Bioorg Med Chem Lett, 2012, 22: 6368-6372 | 0.93 nM |
| 4MXC | Homo sapiens | 2014-10-15 | X-RAY | 1.63 Å | ACS Med Chem Lett. 2014, 5: 673-678 | 6.7 nM |

**Supplementary Table 3.** Pharmacophore summary

| **VEGFR-2 Pharmacophore** | **TP** | **n** | **EF** |  | **c-Met Pharmacophore** | **TP** | **n** | **EF** |
| --- | --- | --- | --- | --- | --- | --- | --- | --- |
| 1Y6B 01 | 25 | 197 | 1.67 |  | 3CD8 01 | 25 | 199 | 2.01 |
| 1Y6B 02 | 25 | 233 | 1.41 |  | 3CD8 02 | 25 | 200 | 2 |
| 1Y6B 03 | 25 | 299 | 1.26 |  | 3CD8 03 | 25 | 227 | 1.76 |
| 1Y6B 04 | 25 | 271 | 1.18 |  | 3CD8 04 | 25 | 268 | 1.49 |
| 1Y6B 05 | 25 | 292 | 1.19 |  | 3CD8 05 | 25 | 274 | 1.46 |
| 1Y6B 06 | 25 | 262 | 1.33 |  | 3CD8 06 | 25 | 269 | 1.49 |
| 1YWN 01 | 9 | 23 | 6.26 |  | 3CD8 07 | 25 | 250 | 1.6 |
| 1YWN 02 | 7 | 17 | 6.59 |  | 3CD8 08 | 25 | 266 | 1.50 |
| 1YWN 03 | 7 | 15 | 7.47 |  | 3CD8 09 | 25 | 284 | 1.41 |
| 1YWN 04 | 8 | 23 | 5.57 |  | 3CD8 10 | 25 | 263 | 1.52 |
| 1YWN 05 | 10 | 27 | 5.93 |  | 3EFK 01 | 23 | 24 | 15.3 |
| 1YWN 06 | 9 | 39 | 3.69 |  | 3EFK 02 | 18 | 45 | 6.4 |
| 1YWN 07 | 8 | 19 | 6.74 |  | 3EFK 03 | 16 | 28 | 9.14 |
| 1YWN 08 | 5 | 43 | 1.86 |  | 3EFK 04 | 21 | 77 | 4.36 |
| 1YWN 09 | 6 | 34 | 2.82 |  | 3EFK 05 | 17 | 33 | 8.24 |
| 1YWN 10 | 9 | 22 | 6.55 |  | 3EFK 06 | 22 | 76 | 4.63 |
| 2OH4 01 | 22 | 23 | 15.3 |  | 3EFK 07 | 21 | 61 | 5.51 |
| 2OH4 02 | 17 | 20 | 13.6 |  | 3EFK 08 | 22 | 54 | 7.33 |
| 2OH4 03 | 11 | 15 | 11.7 |  | 3EFK 09 | 22 | 64 | 5.5 |
| 2OH4 04 | 12 | 18 | 10.7 |  | 3EFK 10 | 21 | 38 | 8.84 |
| 2OH4 05 | 11 | 21 | 8.4 |  | 3VW8 01 | 22 | 65 | 5.42 |
| 2OH4 06 | 9 | 12 | 12 |  | 3VW8 02 | 20 | 68 | 4.71 |
| 2OH4 07 | 11 | 27 | 6.5 |  | 3VW8 03 | 21 | 66 | 5.09 |
| 2OH4 08 | 13 | 30 | 6.9 |  | 3VW8 04 | 22 | 103 | 3.42 |
| 2OH4 09 | 20 | 40 | 8 |  | 3VW8 05 | 18 | 58 | 4.97 |
| 2OH4 10 | 18 | 20 | 14.4 |  | 3VW8 06 | 18 | 47 | 6.13 |
| 2QU6 01 | 19 | 26 | 11.69 |  | 3VW8 07 | 23 | 111 | 3.32 |
| 2QU6 02 | 23 | 50 | 7.36 |  | 3VW8 08 | 22 | 82 | 4.29 |
| 2QU6 03 | 19 | 31 | 9.81 |  | 3VW8 09 | 22 | 73 | 4.82 |
| 2QU6 04 | 22 | 39 | 9.03 |  | 3VW8 10 | 23 | 98 | 3.74 |
| 2QU6 05 | 23 | 43 | 8.56 |  | 3ZZE 01 | 25 | 222 | 1.80 |
| 2QU6 06 | 24 | 68 | 5.65 |  | 3ZZE 02 | 25 | 286 | 1.40 |
| 2QU6 07 | 23 | 46 | 8 |  | 3ZZE 03 | 25 | 287 | 1.39 |
| 2QU6 08 | 25 | 65 | 6.15 |  | 3ZZE 04 | 25 | 308 | 1.30 |
| 2QU6 09 | 23 | 44 | 8.36 |  | 3ZZE 05 | 25 | 313 | 1.28 |
| 2QU6 10 | 23 | 49 |  |  | 3ZZE 06 | 25 | 314 | 1.27 |
| 3BE2 01 | 6 | 31 | 3.10 |  | 4DEI 01 | 24 | 115 | 3.34 |
| 3BE2 02 | 6 | 15 | 6.40 |  | 4DEI 02 | 25 | 213 | 1.88 |
| 3BE2 03 | 10 | 15 | 10.67 |  | 4DEI 03 | 25 | 212 | 1.89 |
| 3BE2 04 | 11 | 21 | 8.38 |  | 4DEI 04 | 25 | 210 | 1.90 |
| 3BE2 05 | 9 | 24 | 6 |  | 4DEI 05 | 25 | 184 | 2.17 |
| 3BE2 06 | 8 | 10 | 12.8 |  | 4DEI 06 | 24 | 205 | 1.87 |
| 3BE2 07 | 11 | 19 | 9.26 |  | 4DEI 07 | 25 | 205 | 1.95 |
| 3BE2 08 | 15 | 32 | 7.5 |  | 4DEI 08 | 25 | 303 | 1.32 |
| 3BE2 09 | 18 | 30 | 9.6 |  | 4DEI 09 | 25 | 302 | 1.32 |
| 3BE2 10 | 14 | 37 | 6.05 |  | 4DEI 10 | 25 | 305 | 1.31 |
| 3CJF 01 | 25 | 239 | 1.67 |  | 4EEV 01 | 19 | 46 | 6.61 |
| 3CJF 02 | 25 | 283 | 1.41 |  | 4EEV 02 | 16 | 32 | 8 |
| 3CJF 03 | 25 | 318 | 1.26 |  | 4EEV 03 | 19 | 50 | 6.08 |
| 3CJF 04 | 25 | 339 | 1.18 |  | 4EEV 04 | 21 | 103 | 3.26 |
| 3CJF 05 | 25 | 335 | 1.19 |  | 4EEV 05 | 21 | 49 | 6.86 |
| 3CJF 06 | 25 | 301 | 1.33 |  | 4EEV 06 | 17 | 40 | 6.8 |
| 3U6J 01 | 23 | 76 | 4.84 |  | 4EEV 07 | 11 | 21 | 8.38 |
| 3U6J 02 | 22 | 66 | 5.33 |  | 4EEV 08 | 18 | 44 | 6.55 |
| 3U6J 03 | 12 | 27 | 7.1 |  | 4EEV 09 | 13 | 25 | 8.32 |
| 3U6J 04 | 8 | 17 | 7.53 |  | 4EEV 10 | 21 | 94 | 3.57 |
| 3U6J 05 | 14 | 39 | 5.74 |  | 4GG5 01 | 16 | 83 | 3.08 |
| 3U6J 06 | 5 | 8 | 10 |  | 4GG5 02 | 14 | 77 | 2.91 |
| 3U6J 07 | 8 | 17 | 7.53 |  | 4GG5 03 | 21 | 91 | 3.69 |
| 3U6J 08 | 8 | 13 | 9.85 |  | 4GG5 04 | 16 | 80 | 3.2 |
| 3U6J 09 | 13 | 44 | 4.73 |  | 4GG5 05 | 18 | 102 | 2.82 |
| 3U6J 10 | 14 | 33 | 6.79 |  | 4GG5 06 | 15 | 68 | 3.53 |
| 3VHE 01 | 20 | 36 | 8.89 |  | 4GG5 07 | 15 | 65 | 3.69 |
| 3VHE 02 | 8 | 15 | 8.53 |  | 4GG5 08 | 7 | 41 | 2.73 |
| 3VHE 03 | 6 | 13 | 7.38 |  | 4GG5 09 | 20 | 99 | 3.23 |
| 3VHE 04 | 10 | 25 | 6.4 |  | 4GG5 10 | 16 | 69 | 3.71 |
| 3VHE 05 | 7 | 14 | 8 |  | 4MXC 01 | 5 | 44 | 1.82 |
| 3VHE 06 | 8 | 18 | 7.11 |  | 4MXC 02 | 3 | 26 | 1.85 |
| 3VHE 07 | 10 | 39 | 4.10 |  | 4MXC 03 | 1 | 8 | 2 |
| 3VHE 08 | 11 | 23 | 7.65 |  | 4MXC 04 | 6 | 59 | 1.63 |
| 3VHE 09 | 15 | 48 | 5 |  | 4MXC 05 | 4 | 28 | 2.29 |
| 3VHE 10 | 9 | 15 | 9.6 |  | 4MXC 06 | 4 | 35 | 1.83 |
| 3VNT 01 | 22 | 57 | 6.18 |  | 4MXC 07 | 10 | 33 | 4.85 |
| 3VNT 02 | 20 | 65 | 4.92 |  | 4MXC 08 | 3 | 11 | 4.36 |
| 3VNT 03 | 23 | 52 | 7.08 |  | 4MXC 09 | 9 | 25 | 5.76 |
| 3VNT 04 | 20 | 31 | 10.32 |  | 4MXC 10 | 3 | 18 | 2.67 |
| 3VNT 05 | 21 | 39 | 8.62 |  |  |  |  |  |
| 3VNT 06 | 20 | 25 | 12.8 |  |  |  |  |  |
| 3VNT 07 | 24 | 76 | 5.05 |  |  |  |  |  |
| 3VNT 08 | 22 | 34 | 10.35 |  |  |  |  |  |
| 3VNT 09 | 11 | 20 | 8.8 |  |  |  |  |  |
| 3VNT 10 | 22 | 43 | 8.19 |  |  |  |  |  |
| 4ASE 01 | 11 | 21 | 8.38 |  |  |  |  |  |
| 4ASE 02 | 7 | 14 | 8 |  |  |  |  |  |
| 4ASE 03 | 20 | 29 | 11.03 |  |  |  |  |  |
| 4ASE 04 | 14 | 22 | 10.18 |  |  |  |  |  |
| 4ASE 05 | 6 | 19 | 5.05 |  |  |  |  |  |
| 4ASE 06 | 18 | 27 | 5.05 |  |  |  |  |  |
| 4ASE 07 | 21 | 28 | 12 |  |  |  |  |  |
| 4ASE 08 | 10 | 18 | 8.89 |  |  |  |  |  |
| 4ASE 09 | 15 | 31 | 7.74 |  |  |  |  |  |
| 4ASE 10 | 7 | 19 | 5.89 |  |  |  |  |  |

**Supplementary Table 4.** The virtual screening results of 18 compounds.

| No. | Compound ID | Structure | VEGFR-2 (2OH4) | | | c-Met (3EFK) | | |
| --- | --- | --- | --- | --- | --- | --- | --- | --- |
|  |  |  | LibDock  Score | -CDOCKER_INTERACTION  _ENERGY  (kcal⋅mol^-1^) | -CDOCKER_ ENERGY  (kcal⋅mol^-1^) | LibDock  Score | -CDOCKER_INTERACTION  _ENERGY  (kcal⋅mol^-1^) | -CDOCKER_ ENERGY  (kcal⋅mol^-1^) |
| 1 | 17924 |  | 157.606 | 67.3681 | 47.2365 | 157.157 | 66.4773 | 52.1612 |
| 2 | 4312 |  | 149.05 | 63.5957 | 46.3905 | 159.177 | 65.316 | 52.1412 |
| 3 | 13461 |  | 157.157 | 63.4773 | 52.1612 | 157.606 | 64.3681 | 47.2365 |
| 4 | 7682 |  | 160.721 | 63.2367 | 45.9686 | 162.58 | 61.1405 | 46.6158 |
| 5 | 11050 |  | 144.960 | 63.1134 | 7.12945 | 148.423 | 62.4733 | 8.71651 |
| 6 | 11693 |  | 154.448 | 63.0864 | 50.9954 | 160.273 | 60.704 | 48.8989 |
| 7 | 5748 |  | 159.956 | 62.6834 | 50.9773 | 145.294 | 60.9212 | 36.9729 |
| 8 | 9973 |  | 155.167 | 62.0747 | 50.0011 | 149.656 | 62.2614 | 36.6803 |
| 9 | 20083 |  | 154.341 | 62.0611 | 42.3287 | 143.973 | 62.9498 | 34.0784 |
| 10 | 1146 |  | 152.754 | 61.9899 | 16.2527 | 156.206 | 62.7612 | 22.6084 |
| 11 | 1348 |  | 158.721 | 61.2725 | 52.046 | 150.573 | 60.926 | 48.4823 |
| 12 | 6493 |  | 154.054 | 61.182 | 14.8072 | 153.621 | 60.0131 | 12.1707 |
| 13 | 18850 |  | 146.742 | 61.1609 | 24.1528 | 166.541 | 63.067 | 27.7422 |
| 14 | 4552 |  | 147.598 | 60.6532 | 27.5826 | 154.781 | 62.6015 | 40.6259 |
| 15 | 1428 |  | 152.792 | 60.65 | 20.4077 | 152.421 | 62.3414 | 20.3564 |
| 16 | 21167 |  | 151.754 | 60.2846 | 41.1227 | 144.876 | 61.3967 | 37.3281 |
| 17 | 10463 |  | 142.530 | 60.2331 | 28.1983 | 169.605 | 63.5136 | 48.0817 |
| 18 | 8512 |  | 151.607 | 60.2255 | 25.6783 | 157.884 | 62.4145 | 26.8013 |

**Supplementary Table 5.** Average, minimum and maximum values of key MD simulations evaluations

| Protein | VEGFR-2 | | | c-MET | | |
| --- | --- | --- | --- | --- | --- | --- |
| Parameter | GIG | Compound  17924 | Compound  4312 | MT4 | Compound  17924 | Compound  4312 |
| Protein backbone atoms RMSD (nm) | | | | | | |
| Average^*^ | 0.3635  (0.1344) | 0.3027 (0.0558) | 0.2972  (0.0417) | 0.3568  (0.0614) | 0.2834  (0.0327) | 0.2498  (0.0420) |
| Maximum | 0.6334 | 0.4442 | 0.4462 | 0.5079 | 0.3813 | 0.3901 |
| Minimum | 0.0831 | 0.0806 | 0.0805 | 0.0998 | 0.0945 | 0.0920 |
| RMSF (nm) | | | | | | |
| Average^*^ | 0.1901  (0.1859) | 0.1478  (0.1485) | 0.1368  (0.0999) | 0.1771  (0.1363) | 0.1550  (0.0859) | 0.1618  (0.1069) |
| Maximum | 1.2468 | 1.3657 | 0.5992 | 1.1249 | 0.4782 | 0.9601 |
| Minimum | 0.0575 | 0.0428 | 0.0427 | 0.0523 | 0.0494 | 0.0479 |
| Rg (nm) | | | | | | |
| Average^*^ | 2.0873  (0.0251) | 2.0598  (0.0176) | 2.0628  (0.0136) | 2.0590  (0.0243) | 1.9972  (0.0144) | 2.0426  (0.0186) |
| Maximum | 2.1692 | 2.1250 | 2.1281 | 2.1421 | 2.0449 | 2.11312 |
| Minimum | 2.0275 | 2.0150 | 2.0223 | 1.9727 | 1.9482 | 1.98132 |
